# P-295. Risk Factors for Mortality in Patients with Klebsiella pneumoniae Carbapenemase (KPC)-Producing Organisms Treated with Ceftazidime/Avibactam: A Systematic Review and Meta-analysis

**DOI:** 10.1093/ofid/ofae631.498

**Published:** 2025-01-29

**Authors:** Ruhul Munshi, Wilmer Salazar, Farah Taufiq, Tanjina Kauser, Mohammad Alam, Paulette Pinargote

**Affiliations:** LSU Health Shreveport, Shreveport, Louisiana; Universidad Catolica Santiago de Guayaquil, Shreveport, Louisiana; Mercy Health St. Elizabeth, Youngstown, Ohio; Tulane University School of Medicine, New Orleans, Louisiana; Louisiana State University Health Sciences Center, Shreveport, LA, USA, shreveport, Louisiana; Louisiana State University Health Services Center Shreveport, shreveport, Louisiana

## Abstract

**Background:**

Klebsiella pneumoniae carbapenemase (KPC)- producing organisms is a leading cause of mortality in hospitalized patients, potentially due to the emergence of resistance to novel antibiotics and limited treatment options. This meta-analysis aimed to elucidate the 30-day mortality rate and characterize risk factors associated with death in patients with KPC infections treated with ceftazidime-avibactam (CTZ-AVM).

**Figure 1:**
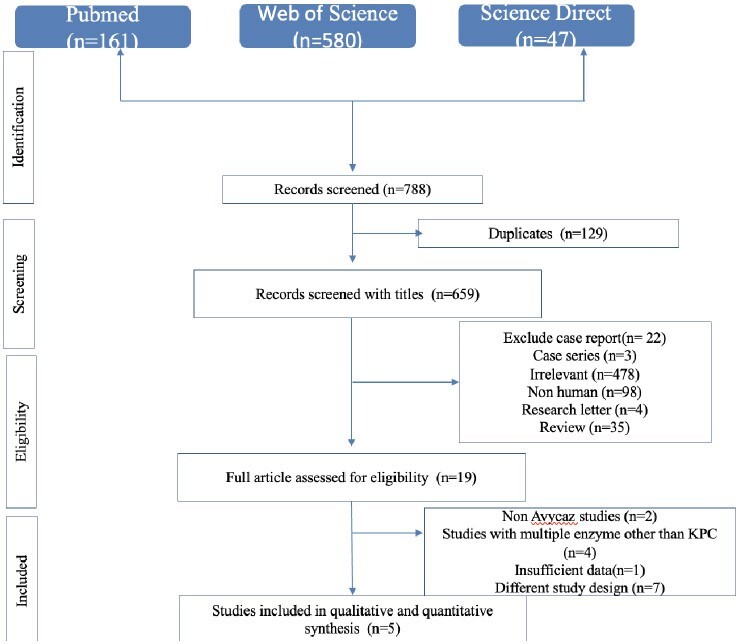
PRISMA flow diagram of studies selection

**Methods:**

Our meta-analysis was conducted in alignment with the Preferred Reporting Items for Systematic Reviews and Meta-Analyses (PRISMA) guidelines. The search terms used were “KPC” or “Klebsiella pneumoniae carbapenemase” in conjunction with “Avycaz” or “Ceftazidime/Avibactam”. Our analysis focused on mortality rate, risk factors, comorbidities, sites of infection, clinical outcomes, and treatment interventions. The primary endpoint was 30-day mortality.

**Figure 2:**
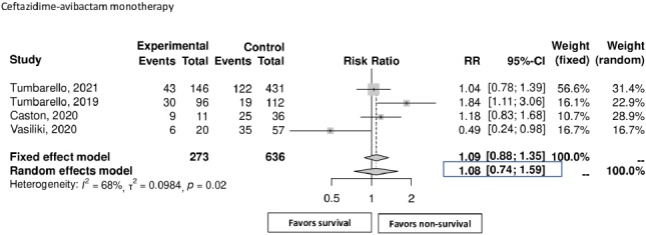
Mortality rate of ceftazidime-avibactam monotherapy

**Results:**

Analysis of five observational studies with 1011 patients revealed an overall 30-day mortality rate of 33% (95% CI: 24%-44%). Mean duration of therapy was 14 days, 72% were male. Patients with comorbidities such as COPD (OR 1.54 [95% CI 1.04-2.29]), cerebrovascular accidents/dementia (OR 1.73 [95% CI 1.17-2.58]), hematologic malignancy (OR 2.01 [95% CI 1.24-3.25]), and neutropenia (OR 3.68 [95% CI 1.98-6.84]) exhibited significant higher mortality rates. Patients diagnosed with pneumonia were found to have a 77% increased risk of death (OR 1.77 [95% CI 1.15-2.72]), while septic shock effectively doubled the mortality risk (OR 2.22 [95% CI 1.82-2.70]). Monotherapy or combination therapy did not significantly influence the 30-day mortality rate.

**Figure 3:**
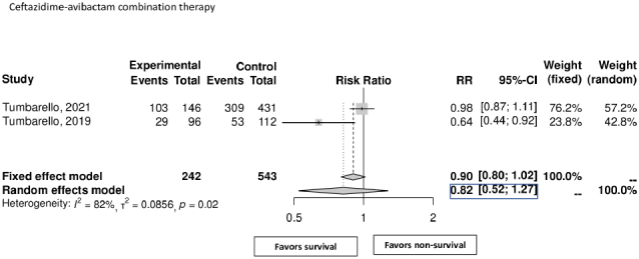
Mortality rate of ceftazidime-avibactam combination therapy

**Conclusion:**

Our meta-analysis suggests that ceftazidime-avibactam monotherapy and combination therapy exhibit similar efficacy in terms of 30-day mortality in patients with infection by KPC-producing organisms. Mortality rates are impacted by different comorbidities, which need to be taken in consideration when managing these infections.

**Disclosures:**

**All Authors**: No reported disclosures

